# Metabolic Response to Androgen Deprivation Therapy of Prostate Cancer

**DOI:** 10.3390/cancers16111991

**Published:** 2024-05-24

**Authors:** Yubin Chen, Pao-Hwa Lin, Stephen J. Freedland, Jen-Tsan Chi

**Affiliations:** 1 Department of Molecular Genetics and Microbiology, Duke University, Durham, NC 27708, USA; yubin.chen@duke.edu; 2 Center of Applied Genomic Technologies, Duke University, Durham, NC 27708, USA; 3 Department of Medicine, Duke University, Durham, NC 27708, USA; pao.hwa.lin@duke.edu; 4 Center for Integrated Research in Cancer and Lifestyle, Cedars-Sinai, Los Angeles, CA 90048, USA; stephen.freedland@cshs.org; 5 Durham VA Medical Center, Durham, NC 27708, USA

**Keywords:** prostate cancer, ADT, metabolic response, diet

## Abstract

**Simple Summary:**

Prostate cancer is the most common cancer in American men, aside from skin cancer, and it is the second deadliest. For patients with advanced or metastatic disease, systemic therapies are warranted. The backbone of these systemic therapies is androgen deprivation therapy (ADT). While providing effective tumor control, ADT can have significant side effects, especially problematic for the many overweight or obese men who are at higher risk for aggressive cancer. Our review investigates how lifestyle changes, particularly a low- carbohydrate diet, might reduce the unwanted metabolic effects of ADT. We specifically discuss two clinical trials, Carbohydrate and Prostate Study 1 (CAPS1) and Carbohydrate and Prostate Study 2 (CAPS2), examining this diet’s impact on metabolic issues and cancer progression. Additionally, we explore how ADT might influence metabolism, aiming to improve patient outcomes by balancing treatment efficacy with quality of life.

**Abstract:**

Prostate cancer (PC) stands as the most frequently diagnosed non-skin cancer and ranks as the second highest cause of cancer-related deaths among men in the United States. For those facing non-metastatic PC necessitating intervention, solely local treatments may not suffice, leading to a possible transition toward systemic therapies, including androgen deprivation therapy (ADT), chemotherapy, and therapies targeting androgen. Yet, these systemic treatments often bring about considerable adverse effects. Additionally, it is observed that overweight men are at a higher risk of developing aggressive forms of PC, advancing to metastatic stages, and succumbing to the disease. Consequently, there is a pressing demand for new treatment options that carry fewer side effects and enhance the current standard treatments, particularly for the majority of American men who are overweight or obese. In this article, we will review the metabolic response to ADT and how lifestyle modulation can mitigate these ADT-associated metabolic responses with a particular focus on the two clinical trials, Carbohydrate and Prostate Study 1 (CAPS1) and Carbohydrate and Prostate Study 2 (CAPS2), which tested the effects of low-carbohydrate diets on the metabolic side effects of ADT and PC progression, respectively. Furthermore, we will summarize the findings of serum metabolomic studies to elucidate the potential mechanisms by which ADT and low-carbohydrate diets can affect the metabolic response to mitigate the metabolic side effects while maximizing therapeutic efficacy.

## 1. Introduction

Prostate cancer (PC) is the most common non-cutaneous cancer among American men. In 2024, it is projected that 299,010 American men will be diagnosed with PC, and it is expected to lead to 35,250 deaths. The incidence rate of PC has increased annually by 3% from 2014 to 2019, and PC is the second leading cause of cancer death among men in the U.S. [1]. Therefore, PC is one of the most important cancers that needs significant attention to improve the outcomes. Classically defined risk factors are older age, obesity, ethnicity, and family history [2]. Other studies clearly show a link between obesity and PC death, if not always with PC incidence [3,4]. Moreover, it has been widely postulated that a Western diet may promote PC progression [5]. Animal studies revealed that high omega-6 fat increased tumor growth while low fat intake inhibited the growth of tumors [6,7].

## 2. Androgen Deprivation Therapy for Prostate Cancer

The standard treatment for advanced and metastatic PC is hormone suppression through androgen deprivation therapy (ADT), which specifically targets androgen receptors and androgen signaling [8,9,10]. While newer treatment options are often added to ADT, they all build upon a backbone of ADT. Since androgen signaling plays an important role in the progression of PC, and testosterone is the primary and most well-known androgen that can stimulate PC growth [10], it is reasonable that ADT is a pivotal therapeutic strategy. ADT functions by reducing or inhibiting androgens’ signaling through surgical interventions like orchiectomy, which directly depletes testosterone by removing its primary source. More commonly, ADT is accomplished by pharmacological means via several approaches. For example, testosterone production can be reduced through feedback mechanisms via either gonadotropin-releasing hormone (GnRH) agonists (e.g., leuprolide) or antagonists (e.g., degarelix). GnRH agonists (e.g., leuprolide) work by initially inducing a surge in luteinizing hormone (LH) and follicle-stimulating hormone (FSH) from the pituitary gland, causing a temporary rise in testosterone. However, continued use leads to the pituitary gland becoming desensitized to GnRH, which decreases LH and FSH secretion and subsequently lowers testosterone production [11,12].

Apart from GnRH agonists and antagonists, it is also possible to antagonize androgen receptor (AR) function through anti-androgen drugs (e.g., bicalutamide, enzalutamide, apalutamide, darolutamide) [13]. ADT’s applications in PC are quite broad, spanning from neoadjuvant or adjuvant to enhanced radiation therapy as well as for advanced-stage and metastatic PC. However, while ADT’s benefits are undeniable, it brings along a spectrum of side effects. Another major limitation is that ADT only remains effective for limited durations, after which the tumors start to grow again, a condition known as castration-resistant prostate cancer (CRPC). Emerging research indicates that the pentose phosphate pathway (PPP), particularly through its rate-limiting enzyme glucose-6-phosphate dehydrogenase (G6PD), plays a critical role in the progression of prostate cancer to bone metastases, correlating with a poorer prognosis [14]. The suppression of G6PD has been observed to hinder the growth and migration of prostate cancer cells, highlighting its potential as a therapeutic target. Additionally, the bone microenvironment drives the up-regulation of G6PD via interleukin-6 (IL-6) [14]. Notably, the expression of G6PD is up-regulated under androgen-deprived conditions commonly induced by first-line ADT for metastatic PC [14].

## 3. Metabolic Side Effects of ADT

While the clinical benefits of ADT are substantial, the therapy is not without its drawbacks, particularly in terms of metabolic health. For instance, ADT can increase serum total cholesterol (both LDL and HDL), risk of insulin resistance, and diabetes [15]. Given the extended survival of PC patients due to ADT and other effective treatments, the management of metabolic side effects associated with ADT is essential, as these side effects can significantly counteract the benefits of ADT, thereby reducing mortality and increasing other disease risks. Therefore, a thorough understanding and management of the metabolic responses to ADT are crucial. Chief among these metabolic perturbations is insulin resistance, which not only predisposes patients to the development of type 2 diabetes mellitus but also develops hyperglycemia, thus fostering a pro-atherogenic milieu, elevating the risk of cardiovascular events [16,17]. Moreover, diets with a high glycemic index have been linked to heightened risks of cardiovascular disease and mortality [18]. In addition, ADT also altered lipid metabolisms, including an increase in low-density lipoprotein (LDL), high-density lipoprotein (HDL), and total cholesterol levels. Together, such insulin-resistant states and worse lipid profiles further compound the cardiovascular risk profile. However, it is unclear the degree to which the type of ADT and/or preexisting cardiovascular morbidity predicts the risk of a future cardiovascular event in this population [19]. Furthermore, most patients with PC are obese/overweight, which further exaggerates the impact of these metabolic side effects.

Other than these metabolic side effects, there is also a perturbation of bone metabolism. Androgens, via aromatization to estrogens, are well recognized to increase bone density [20]. As such, ADT leads to a decline in bone mineral density (BMD), predisposing patients to osteopenia and osteoporosis, thus heightening the risk of fractures and associated morbidities [21]. In addition, bone density can be regulated by interleukin 6 (IL6), and the level of IL-6 can predict bone loss and resorption [22]. Transgenic mice engineered to overproduce IL-6 lead to increased circulating levels of IL-6, which, in turn, stimulates the formation of osteoclasts and low bone mass [23]. Additionally, ADT can reduce lean mass and increase weight as well as the percentage of fat body mass [17,24]. The loss of muscle mass can particularly contribute to frailty, reduced functional status, and compromised physical performance. Recent research indicated that the gut microbiota composition in men receiving ADT is altered [25]. This disruption in gut microbiota, characterized by reduced bacterial diversity, has been linked to various health conditions, including inflammatory bowel disease, obesity, neurological disorders, and heightened frailty—some of which also correspond to the side effects associated with ADT [26,27]. Furthermore, long-term ADT experiences a rise in certain gut microbiota genera that are associated with testosterone production and potentially harmful bacteria [28]. It is imperative to recognize that these metabolic side effects are not mere bystanders but actively interact, potentially creating a vicious cycle. For instance, increased adiposity can further exacerbate insulin resistance and increase skeletal fragility due to bone mineral density loss.

In addition to the effects on the metabolic and microbiome, ADT can alter the brain metabolism associated with verbal memory, spatial abilities, and mood swings, including medial thalamus bilaterally, cerebellum, and posterior cingulate [29]. The areas of the brain affected by ADT coincide with those areas that experience metabolic reduction in the early stages of Alzheimer’s disease and individuals with diabetes [29]. The cumulative nature of these metabolic side effects underscores the need for meticulous monitoring, early intervention, and comprehensive management strategies for patients undergoing ADT, encompassing dietary guidance, physical activity recommendations, and targeted pharmacotherapies when appropriate [30].

Our previous metabolomic investigation revealed that ADT elicits significant alterations in systemic metabolism over a period extending to six months [31]. Initially, at the three-month juncture, ADT predominantly affected the serum concentrations of long-chain acyl-carnitines, notably hydroxy myristoyl-carnitine, malonyl-carnitine, and several other derivatives. Concurrently, we observed decreased levels of androsterone sulfate, 3-hydroxybutyric acid, and indoleacetic acid (IAA), the latter of which is a tryptophan-derived metabolite originating from the gut microbiome [32]. Progressing to the six-month threshold, the metabolic landscape further evolved. Notably, we detected an increase in compounds such as dihydroxycholestanoyl taurine, AMP, *N*-acetyl-glucosamine-1-phosphate, mevalonate-5-phosphate, and 2-hydro-d-gluconate [31]. This phase also saw a continuation of the diminishing trend in several long-chain acylcarnitines, reaffirming the consistent impact of ADT on lipid metabolism pathways. Moreover, this extended duration of ADT further reduced the plasma concentrations of organic acids, including oxalic acid, glycolic acid, and nonanoic acid.

In parallel, structural lipid analyses juxtaposing ADT-treated and untreated prostate cancer cohorts emphasized a marked and sustained decrease in 3-hydroxybutyric acid [31]. This finding, along with the reductions in various long-chain acyl-carnitines and other fatty acid metabolites, underscores the potential of ADT to substantially modulate ketogenesis and fatty acid metabolism. Intriguingly, free carnitine levels remained unaltered, indicating a specific targeted effect of ADT rather than a broad suppression of lipid-associated metabolites.

## 4. Rationale for Low-Carbohydrate Diets (LCDs) during ADT Treatment of Prostate Cancer Patients—As Observed in Two Clinical Trials, CAPS1 and CAPS2

Common adverse metabolic effects of ADT in PC patients, such as weight gain, increased adiposity, and insulin resistance, necessitate dietary strategies that could mitigate these consequences. Carbohydrates are the primary dietary macronutrient responsible for elevating postprandial serum glucose and insulin levels [33]. Low-carbohydrate diets (LCDs) are emerging as a significant intervention due to their proven efficacy in managing diabetes and promoting weight loss. Typically, a Western diet derives 40–60% of its calories from carbohydrates, whereas LCDs contain fewer than 20% of calories from this macronutrient [34]. Both LCDs and ketogenic diets (a form of very low-carbohydrate diet) have demonstrated efficacy in managing diabetes and facilitating weight loss [35,36,37]. Given the well-appreciated metabolic benefit associated with LCDs, there is a strong rationale to test whether LCDs and other lifestyle interventions can mitigate these ADT-associated metabolic adverse effects. Such a possibility was supported by experimental evidence in animal models. For example, in comparative studies of PC xenografts, it was observed that an LCD led to extended survival rates [38,39] and inhibited the growth of prostate cancer in a genetically modified mouse model [40], even in the absence of any reduction in body weight, as evidenced by our findings from two separate studies. These data further supported the strong scientific rationale for testing LCDs in PC.

In order to ascertain the clinical advantages of a low-carbohydrate diet (LCD) in individuals afflicted with prostate cancer (PC), our team initiated two clinical investigations: Carbohydrate and Prostate Study 1 (CAPS1) and Carbohydrate and Prostate Study 2 (CAPS2). The CAPS1 investigation was designed to assess the consequences of a six-month LCD on insulin resistance amongst PC patients commencing androgen deprivation therapy. Conversely, CAPS2 [41] was structured to evaluate the efficacy of a six-month LCD in mitigating the proliferation of PC in individuals presenting with biochemical recurrence post-therapeutic intervention. Additionally, serum metabolomic analysis was performed on specimens procured from both the intervention and control cohorts within each respective study.

The results from both CAPS1 and CAPS2 have been published [41]. In CAPS1, three months of intervention with LCD significantly reduced body weight, insulin resistance, and other metabolic parameters, including improved HbA1c, triglyceride, and high-density lipoprotein (HDL) levels [41]. However, only some of the metabolic benefits (weight loss and HDL) persisted at the end of the study (6 months). At six months, other metabolic parameters reversed toward the baseline and became insignificant between the two arms. This may be due to the small number of patients. Nevertheless, these potential benefits of LCD provide a rationale for additional studies and examination.

In contrast to CAPS1, CAPS2 was designed to evaluate the effects of LCD on PC progression after biochemical recurrence (BCR) after local treatments. The PC progression was measured based on the prostate-specific antigen (PSA) doubling time (PSADT), shown to be strongly correlated with death in patients with recurrent PC. Similar to the results in CAPS1, LCD intervention also significantly reduced body weight, increased HDL, and reduced triglycerides and HbA1c, which were significant at 6 months. Interestingly, post hoc analyses were adjusted for the baseline indifferences between the groups. LCD was also associated with a suggested longer PSADT. Even though future larger studies are needed to verify the benefits of an LCD, the encouraging results provide further reasons to pursue the clinical benefits in other settings and larger studies.

## 5. Metabolomic Analysis of CAPS1-ADT in Patients with PC with or without LCD

To grasp the metabolic impact of various interventions in CAPS1, we analyze the serum metabolomic of these patients during ADT with or without LCD. We aim to capture all the metabolic changes using the mass spectrometry (MS)-based metabolomic approaches that can map out the small molecule metabolites present in tumor cells, tissues, blood serum, and urine. Metabolomics, by examining all metabolites, acts as a comprehensive indicator of all preceding biochemical activities triggered by somatic mutations, changes in gene expression, and the proteome throughout the progression and treatment of tumors. The metabolomic analysis also provides an integrated perspective of all biochemical events that are affected by the therapeutic intervention and lifestyle choices [42,43,44]. In PC work by others, such metabolomic analysis identified sarcosine pathways as critical in oncogenesis [45,46]. Other metabolomic studies identified therapeutic targets to treat PC [47,48,49]. While ADT is the standard systemic PC treatment, only a few studies have analyzed the serum metabolome in patients receiving ADT [31,50,51]. For example, one study has found that ADT significantly affected metabolites of androgen steroids, bile acid metabolites, and lipid β- and Ω-oxidation [51]. Other studies identified seven ADT-associated metabolites that reverted to control levels after the cessation of ADT, indicating the reversibility of these metabolic changes [50].

**Figure 1:**
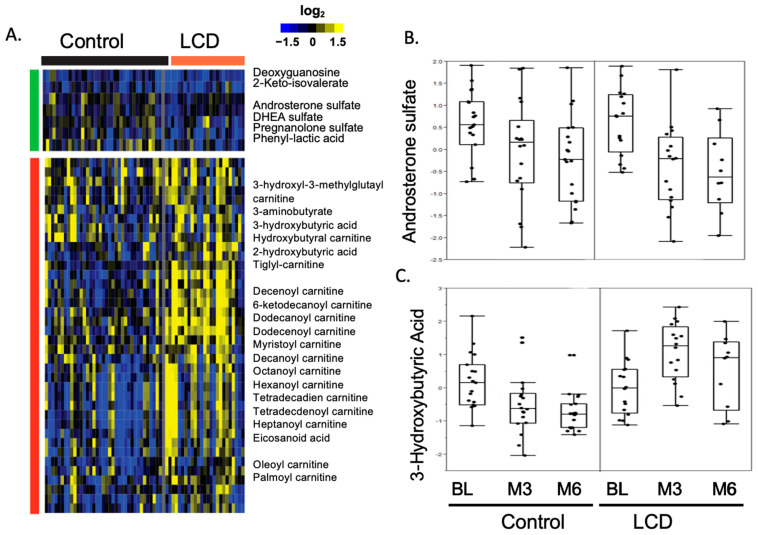
Heatmap showcasing the changes in metabolite levels induced by ADT within the control and LCD groups in the CAPS1 study. (**A**) This heatmap is generated by applying zero-transformation [59] to the changes in metabolite levels from baseline (BL) in response to ADT, organized through hierarchical clustering. Increases are represented by yellow, decreases by blue, and no change is indicated by black. (**B**) ADT decreased androsterone sulfate levels in both the control and LCD groups. (**C**) The ADT-related alterations in 3−hydroxybutyric acid levels at the third and sixth months (M3 and M6) within both control and LCD groups.

Our previous examination of the metabolomic response to ADT involved comprehensive profiling of serum samples obtained from CAPS1 participants, both pre-ADT and after a six-month regimen. In conjunction with this, we correlated these metabolomic findings with clinical blood chemistry data to assess the systemic effects of ADT, including alterations in glucose levels, lipid profiles, and other metabolic health parameters. This innovative approach incorporated lipidomic analysis and integrated it with serum metabolomic data to provide a holistic view of the metabolic shifts in ADT-treated patients. As anticipated, ADT markedly diminished steroid biosynthesis, evident from decreased levels of androsterone sulfate, dehydroepiandrosterone (DHEA) sulfate, and pregnanolone sulfate, thereby confirming the therapeutic aim of lowering androgen levels (Figure 1A,B). Additionally, the naturally occurring steroid hormone DHEA emerges as an uncompetitive inhibitor of G6PD, implicating its role in the metabolic milieu influencing prostate cancer [52]. As a precursor for androgens and estrogens and the most abundant circulating steroid, DHEA is metabolically significant, providing a substantial contribution to the dihydrotestosterone present in the prostate [53]. Rat models of prostate cancer suggest a protective role for DHEA against the development of the disease [54]. But in patients, lower serum levels of DHEA align with more aggressive prostate cancer characteristics, such as a high Gleason score, advanced clinical stage, and notably, a worse prognosis in hormone-naïve prostate cancer and poorer cancer-specific survival in metastatic castration-resistant prostate cancer (mCRPC) patients [55]. These findings suggest that DHEA is a potent uncompetitive inhibitor of G6PD, and a lower level of DHEA in patients might cause upregulation of G6PD and promote tumor growth and metastasis.

Concurrently, a reduction in 3-hydroxybutyric acid observed at both three- and six-month intervals (Figure 1A,C) suggests a suppression of ketogenesis or an elevation in ketone body consumption. Since previous studies have linked heightened β-hydroxybutyrate and certain ketogenic enzymes to prostate cancer progression, the decrease in ketone bodies during ADT may be implicated in the development of insulin resistance [56]. This connection underpins the potential utility of ketogenic diets in ameliorating insulin resistance, possibly through the elevation of ketone bodies to counteract this metabolic complication [57]. Thus, the observed decrease in ketone bodies associated with ADT underscores the rationale behind employing low-carbohydrate diets to reverse adverse metabolic effects induced by ADT.

Furthermore, ADT has a broader impact on metabolism, affecting acyl-carnitine profiles, glucose processing, glycolysis, and metabolites related to the microbiome. Notably, changes in androsterone sulfate—a marker of androgen suppression—were strongly correlated with increased levels of indole-3-carboxaldehyde (ICA), a microbial derivative (refer to Figure 1A,C). ICA has been identified as a stimulator of aryl hydrocarbon receptors in intestinal immune cells, promoting IL-22 production, which plays a role in mitigating gastrointestinal inflammation and protecting against epithelial damage [32,58]. Therefore, an increase in ICA during ADT may contribute to the observed decrease in inflammatory bowel diseases commonly associated with ADT [58].

Next, we applied MS-based serum metabolomic profiling to capture all the metabolic effects of LCD on ADT-induced metabolic changes (Figure 1). As expected, LCD significantly increased the levels of various ketone bodies and reversed the ADT-reduced ketogenesis (Figure 1A,C). Importantly, LCD did not only compromise the ADT-reduced androgens but may slightly enhance the androgen reduction (Figure 1A,B), although the enhancement was not statistically significant. LCDs also counteracted the decrease in ketogenesis and acyl-carnitines caused by ADT and disrupted the association between elevated serum glucose levels and reduced androsterone sulfate, in addition to reversing ADT-mediated changes in glycolysis and amino acid metabolism. Collectively, LCDs effectively counterbalanced many of the metabolic alterations induced by ADT in the serum, potentially alleviating metabolic side effects linked to ADT. These benefits include weight reduction, decreased insulin resistance, and improvements in hemoglobin A1c, HDL cholesterol, and triglyceride levels, as previously detailed in our CAPS1 study. Moreover, the metabolic modifications brought about by LCDs could imply their potential as complementary therapies, contributing to anti-tumor effects. These include the possible reinforcement of androgen suppression, diminished tumor glycolysis, and a reduction in metabolites that suppress the immune system, such as lactate and kynurenine.

## 6. Metabolomic Analysis of CAPS2-LCD in PC Patients with Biochemical Recurrence

In contrast to CAPS1, CAPS2 was designed to evaluate the effects of LCD on the PC progression after biochemical recurrence. The study aimed to determine the impact of a six-month LCD intervention on PC progression, as measured by PSADT. The CAPS2 study showed that LCD does not negatively slow tumor growth and may even lead to a slower PSADT, suggesting delaying PC progression [41]. For the metabolic effects, LCD in the CAPS2 trial resulted in significant weight loss, increased HDL, and decreased triglycerides of the participants, consistent with the metabolic benefits seen in patients of CAPS1.

**Figure 2:**
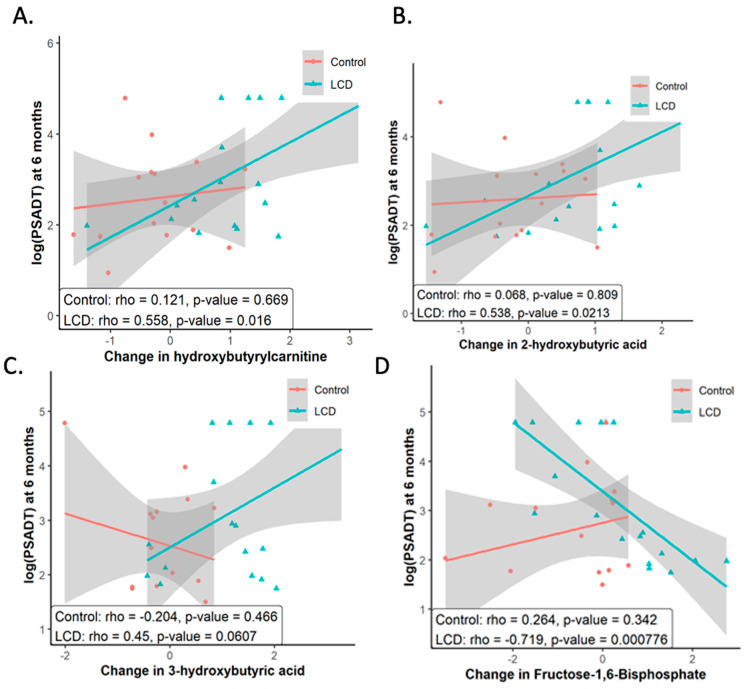
Metabolites that changed under LCD correlated with PSADT among subjects in CAPS2. Higher levels of (**A**) 3-hydroxy-2-methylbutyriuc acid (**B**) hydroxyl-butyryl-carnitine (**C**) 2-hydroxybutyric acid were significantly associated with longer PSADT at Month 6. (**D**) An increase in Fructose 1,6-bisphosphate was associated with shorter PSADT. These figures illustrate the association between alterations in selected metabolites, triggered by LCD with PSADT across both control and LCD study groups. It includes the presentation of statistical significance, depicted through p-values, underscoring the correlation strength between each identified metabolite and PSADT variations.

As a secondary analysis, we analyzed the serum metabolomics and found the elevation in ketone body levels due to the LCD-affirmed adherence to the diet and anticipated ketogenic results. With a significant rise in ketone bodies from the LCD, we analyzed how this increase correlated with changes in the PSADT. Our attention was particularly on the Month 6 timepoint since PSADT calculations utilized prostate-specific antigen (PSA) readings from the start of the study baseline (BL), Month 3, and Month 6 post-randomization. We assessed metabolites whose variations throughout the study period were linked with PSADT changes. Longer PSADT was significantly associated with higher 3-hydroxy-2-methylbutyriuc acid (Figure 2A), hydroxyl-butyryl-carnitine (Figure 2B), and 2-hydroxybutyric acid (Figure 2C) at Month 6. The observed positive relationship between the rise in ketone bodies and extended PSADT suggests the hypothesis that enhanced ketogenesis from an LCD might be linked to slower tumor progression. Although the LCD did not uniformly decrease all metabolites associated with glycolysis, a shorter PSADT was found to correlate with an increase in the glycolysis-related metabolite fructose-1,6-bisphosphate (Figure 2D). Fructose 1,6-bisphosphate is a metabolic intermediate in the glycolytic pathway. Reducing glycolysis is an important goal of LCD for cancers [60]. Our findings are consistent with the hypothesis that more prominent reduced glycolysis under LCD is associated with slower PC progression. We previously showed that androgen sulfate, the main driver of PC growth, was most consistently reduced by ADT [31,61]. In this analysis, alterations in androgen sulfate did not show a correlation with PSADT. Collectively, these findings indicate that the association of slower tumor progression and extended PSADT within the LCD group correlates with a notable increase in ketogenesis and tricarboxylic acid (TCA) cycle metabolites, alongside a greater decrease in fructose-1,6-bisphosphate levels, an intermediate metabolite of glycolysis. Such a correlation suggests a connection between PSADT and reduced glycolysis. One study revealed that in the cases of prostate cancer bone metastasis, there is an increase in the levels of glutathione, reactive oxygen species (ROS) detoxification, and the TCA cycle, compared with primary prostate cancer [14].

## 7. Summary of the Metabolomic Analysis of CAPS1 and CAPS2 (Figure 3)

**Figure 3:**
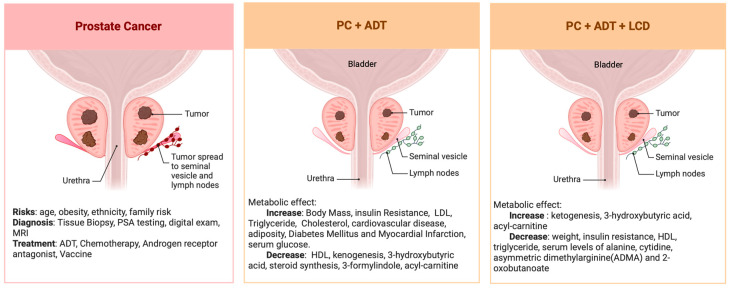
Summary of the serum metabolomic changes in PC undergoing ADT before and after LCD.

Serum metabolomic analysis revealed that ADT significantly reduced both ketogenesis and fatty acid metabolism (Figure 3). Additionally, ADT may influence the microbial synthesis of indoles, which are implicated in immune response pathways. Follow-up studies are imperative to validate these observations and elucidate the precise mechanisms and functional implications of ADT on metabolic balance in PC patients to circumvent potential ADT-related comorbidities. Furthermore, our findings indicate that an LCD might counteract various metabolic disturbances associated with ADT without compromising its therapeutic effectiveness. Intriguingly, there is evidence suggesting that LCD could even potentially amplify the effects of ADT on reducing androgen sulfate levels and decelerating the PSADT. Although preliminary, these insights highlight the possibility of integrating LCD with conventional cancer therapies to potentiate their therapeutic outcomes. This integrative approach warrants further investigation to confirm its efficacy across different oncological treatments.

These metabolomic analyses provide some novel insights into metabolic dysregulations as well as the potential of LCD to correct these metabolic dysregulations. For example, while ADT is expected to lower the levels of all male hormones, we found that androsterone sulfate levels were most notably decreased. In fact, the degrees of the changes in androsterone sulfate may, in the future, be useful as surrogate biomarkers to measure the on-target efficacy of ADT. For example, a strong correlation was initially found between the reduction in androsterone sulfate and levels of glucose and indole-3-carboxaldehyde during ADT. However, this association was disrupted by LCD, suggesting a potential connection by which LCD may mitigate the metabolic dysregulation of ADT. Furthermore, we also found that LCD can modestly enhance the reduction in androsterone sulfate, consistent with the prolonged PSADT. These results may suggest the potential of LCD to possibly delay PC progression through additional androgen lowering.

Another aspect of the unexpected findings is related to the microbiome-related metabolites, as illustrated by indole-3-carboxaldehyde (ICA), a microbiota-derived metabolite. There was a strong correlation between the ADT-reduced androsterone sulfate and alterations in ICA, which activates aryl hydrocarbon receptors on intestinal immune cells, triggering the production of IL-22 [32]. Therefore, ICA plays a crucial role in controlling mucosal reactivity and inflammation, consistent with the observation that ICA treatments can diminish gut inflammation, protect against epithelial damage, prevent bacteria from crossing the intestinal barrier, and lower levels of inflammatory cytokines [58]. Notably, the elevated ICA levels during ADT may alleviate inflammatory bowel diseases (IBDs). However, since an LCD appears to counteract the ADT-induced ICA changes, it might also reduce the protective effects against IBD conferred by ADT. The mechanisms by which ADT and LCD affect the production/consumption of ICA and the resulting impact on mucosal immunity and inflammation remain to be studied in future research.

While this metabolomic analysis suggests some intriguing trends, these results and any conclusions must be viewed in light of several constraints. Primarily, the limited size of our sample could diminish our capability to identify significant differences that hold biological relevance and are potentially crucial. Consequently, our findings should be seen as preliminary, serving to generate hypotheses that necessitate corroboration through more definitive research. The absence of comprehensive metabolic data for all participants might have influenced the equilibrium of our randomization process. Additionally, the constrained size of our datasets complicates the examination of potential variances among patients with diverse clinical profiles, such as varying ethnic backgrounds or tumor grades. It is also important to note that our studies did not allow for the differentiation between the effects of LCD itself and those resulting from weight loss, a distinction that requires further investigation. Lastly, there is a need for subsequent research to link these metabolomic alterations to longer-term health outcomes, including tumor management and the development of metabolic complications like diabetes and cardiovascular disease.

## 8. Potential of Low-Carbohydrate Diets to Mitigate the Metabolic Adverse Effects of ADT

Our analysis has provided strong evidence that LCD may mitigate the metabolic side effects of ADT while not affecting the efficacy of ADT. Such a specific effect is evidenced by the LCD-affected changes in metabolic parameters in the blood chemistry and serum metabolomic analysis. Following LCDs during cancer therapies [62,63] can help manage blood sugar levels and reduce insulin resistance, thereby mitigating metabolic complications like hyperglycemia. Furthermore, LCD may also protect normal cells from the toxic effects of treatments since only normal cells, but not cancer cells with altered metabolism, could utilize ketones for energy. Therefore, cancer cells without the ability to utilize ketone bodies [64,65,66] are at a selective disadvantage in host environments under LCD.

Additionally, LCDs can improve body composition and physical well-being, potentially decreasing treatment-related fatigue by preserving muscle mass. They might also alleviate gastrointestinal distress by altering the gut microbiota and reducing sugar fermentation in the gut. Furthermore, the anti-inflammatory properties of LCD [67,68] could help in reducing the chronic inflammation often exacerbated by cancer treatments, contributing to an overall reduction in treatment-related side effects.

## 9. Potential of Low-Carbohydrate Diets to Enhance the Therapeutic Efficacy

Our results also suggest the potential of LCD to enhance the therapeutic benefits of ADT. Although the small sample size and marginal statistics necessitate further studies with more patients, it is helpful to consider LCD in the context of other cancer therapeutics. The role of LCD in cancer therapeutics is becoming increasingly recognized for its potential to complement and enhance the efficacy of traditional cancer treatments [62,63]. LCD alters the metabolic environment that cancer cells exploit for growth and survival by reducing blood glucose levels and insulin secretion, conditions that promote the proliferation and progression of cancer cells. Such metabolic shift can lead to a state of ketosis, where the body utilizes ketone bodies as an alternative energy source, potentially exerting anti-cancer effects by starving cancer cells of their preferred fuel source, glucose, since cancer cells may lose the ability to utilize ketone bodies.

In the context of chemotherapy, LCD may enhance the sensitivity of cancer cells to chemotherapeutic agents, potentially increasing the efficacy of treatment while simultaneously reducing its side effects. This ketogenesis diet-driven change results in increased tumor sensitivity to chemotherapy and potentially improves therapeutic outcomes [69,70]. A recent study has shown the potential of LCD to render PI3K inhibitors, normally inactive against pancreatic cancer, to become effective in murine Kras-driven pancreatic (KPC) tumors, presumably due to the mitigation of feedback insulin resistance mechanisms [71]. This result suggests that the benefit of LCD and other diets [72] might be tailored to enhance therapies targeting insulin-related pathways.

Ferroptosis is a newly recognized form of cell death and tumor suppression mechanisms with a significantly promising therapeutic approach [73]. Interestingly, inducing ferroptosis is particularly effective for the resistant and recurrent tumor cells [74], including YAP/TAZ-driven resistance [75] to most cancer therapeutics. Therefore, promoting ferroptosis fulfills an unmet need that can target the most difficult tumor cells without effective cancer therapeutics. Ferroptosis is a metabolic progress that involves the depletion of several ferroptosis-protection pathways and metabolites, such as glutathione (GSH) and NADPH, and cysteine serves as an essential precursor for GSH synthesis [73]. NADPH plays a critical role in maintaining redox homeostasis by facilitating the regeneration of glutathione (GSH), which, in turn, supports the antioxidant activity of glutathione peroxidase 4 (GPX4) [76]. Depletion of either NADPH or GSH compromises GPX4 functionality, potentially triggering ferroptosis, a form of programmed cell death driven by iron-dependent lipid peroxidation. It has been observed in prior studies that ADT leads to the upregulation of G6PD, which is pivotal for NADPH production. An increase in G6PD activity will enhance NADPH levels, bolstering cellular defense mechanisms against ROS and offering increased resistance to ferroptosis [14]. Since LCD affects the redox environments of tumor cells, it is unsurprising that LCD has been found to enhance the ferroptosis of cancer cells [77,78]. Consistently, we have found that LCD affects the levels of several cysteine-containing metabolites that play a protective role against ferroptosis [59,79], thus supporting the potential of LCD to enhance ferroptosis.

For immunotherapy, emerging evidence suggests that the composition of the gut microbiota—a factor significantly influenced by diet—can impact the effectiveness of immunotherapies targeting programmed cell death protein 1 (PD-1) and programmed death-ligand 1 (PD-L1) [80]. We and others have shown that LCD can alter the gut microbiome metabolites in a way that suggests a modulated immune system that may enhance the body’s response to immunotherapy. Preliminary findings from studies involving LCD support the notion that manipulating the gut microbiome can convert non-responders to responders, highlighting the potential of dietary strategies to improve immunotherapy outcomes [81,82].

Overall, the integration of LCD into cancer therapeutic protocols offers a promising adjunct strategy to enhance conventional treatments. By targeting the metabolic vulnerabilities of cancer cells and potentially modulating the immune response, LCD could improve treatment efficacy, reduce side effects, and contribute to a more personalized approach to cancer care. However, these need to be tested in randomized trials to confirm such benefits. Moreover, further research is necessary to fully understand the mechanisms involved and to establish evidence-based guidelines for the implementation of dietary interventions in cancer therapy. It is likely the metabolomic analysis of the serum and tumor metabolites during LCD may offer important insights into the mechanisms of response vs. resistance to such therapy-enhancement effects.

## 10. Discussion and Future Direction

ADT would cause several adverse metabolic effects in patients, and our results indicate the potential of LCD to augment the therapeutic benefits of ADT and other cancer treatments and reduce metabolic side effects, though these conclusions are preliminary and require validation through larger-scale studies. There is a pressing need for follow-up research to establish a direct connection between these metabolomic changes and long-term health outcomes, specifically in terms of tumor control and the reduction of metabolic diseases like diabetes and cardiovascular diseases. Future studies should aim to confirm these findings and expand our understanding of how dietary interventions can be strategically employed to enhance cancer therapy and patient well-being.

## 11. Conclusions

In conclusion, prostate cancer remains a significant health challenge, with its prevalence and mortality rate positioning it as a critical focus of oncological research and treatment. As prostate cancer progression is driven by androgens, ADT serves as the cornerstone of systemic treatment for advanced and metastatic prostate cancer. However, the metabolic side effects of ADT, particularly in the context of the high prevalence of overweight and obesity in American men, highlight a need for strategies that can mitigate these metabolic adverse effects to enhance efficacy and improve overall quality of life. Our review emphasizes that lifestyle interventions, such as the adoption of a low-carbohydrate diet, have the potential to alleviate the adverse metabolic consequences of ADT without affecting its efficacy. The findings from the CAPS1 and CAPS2 trials provide compelling evidence that dietary modification could play a crucial role in managing these side effects and perhaps even retard cancer progression. The insights gained from metabolomic studies of these cohorts further our understanding of the intricate mechanisms at play, offering a hopeful prospect for enhancing the effectiveness of existing therapies and improving the overall management of prostate cancer.
